# The most important questions in cancer research and clinical oncology—Question 2–5. Obesity-related cancers: more questions than answers

**DOI:** 10.1186/s40880-017-0185-8

**Published:** 2017-01-31

**Authors:** Ajit Venniyoor

**Affiliations:** National Oncology Center, Muscat, Oman

## Abstract

Obesity is recognized as the second highest risk factor for cancer. The pathogenic mechanisms underlying tobacco-related cancers are well characterized and effective programs have led to a decline in smoking and related cancers, but there is a global epidemic of obesity without a clear understanding of how obesity causes cancer. Obesity is heterogeneous, and approximately 25% of obese individuals remain healthy (metabolically healthy obese, MHO), so which fat deposition (subcutaneous versus visceral, adipose versus ectopic) is “malignant”? What is the mechanism of carcinogenesis? Is it by metabolic dysregulation or chronic inflammation? Through which chemokines/genes/signaling pathways does adipose tissue influence carcinogenesis? Can selective inhibition of these pathways uncouple obesity from cancers? Do all obesity related cancers (ORCs) share a molecular signature? Are there common (over-lapping) genetic loci that make individuals susceptible to obesity, metabolic syndrome, and cancers? Can we identify precursor lesions of ORCs and will early intervention of high risk individuals alter the natural history? It appears unlikely that the obesity epidemic will be controlled anytime soon; answers to these questions will help to reduce the adverse effect of obesity on human condition.

## Introduction

There is a global obesity pandemic. Obese people get more cancers. These are two incontrovertible facts. However, attempts to clarify the mechanistic links between obesity and cancer have raised more questions than answers.

Excess body fat is a major global public health problem. In 2014, the World Health Organization classified 67% of the population of the United States, 63% of the United Kingdom, and 64% of Australia as overweight or obese [1]. The global obesity pandemic is not limited to Western countries; it also affects “poor countries” such as India. Paradoxically, while India is home to the most underweight people in the world (202 million) in 2014, it also has the fifth highest number of obese men (9.8 million) and third highest number of obese women (20 million) [2]. The rate of increase in overweightness and obesity appears to have been highest between 1992 and 2002; although the trend in developed countries has slowed over the last decade, it continues to rise in other regions of the world [3].

Obesity accounts for approximately 5% of new cancers in adults [4], making it the second highest risk factor. Tobacco exposure is the highest risk factor; however, while the biological and natural history of tobacco-related cancers have been elucidated and effective preventive programs have been implemented with positive results, management of obesity-related cancers (ORCs) lags far behind.

The World Cancer Research Fund (WCRF) listed 10 cancers as obesity-related [4], including post-menopausal breast, endometrial, ovarian, advanced prostate, colorectal, renal, pancreatic, liver, and gallbladder cancers, as well as esophageal adenocarcinoma. The WCRF estimated that 28% of gallbladder cancers, 35% of pancreatic cancers, and 35% of esophageal cancers are attributable to obesity. The International Agency for Research on Cancer (IARC) recently released an update on the link between obesity and cancer, adding another eight cancers (cancers of the gastric cardia, liver, gallbladder, pancreas, ovary, and thyroid, as well as meningioma and multiple myeloma) to their previous list of five (cancers of the colon, esophagus, kidney, breast, and uterus) as having sufficient evidence to be called risk factors [5]. Another three (prostate cancer, male breast cancer, and diffuse large B cell lymphoma) were linked with limited evidence. The IARC did not comment on association versus causation.

In the field of adiponcosis, the principal questions that need answers are as follows.

### Question 1

Cancer and obesity are heterogeneous. Which molecular subtypes of cancer are related to or caused by “malignant” obesity?

## Background

Obesity is heterogeneous [6]. Obesity is not always pathogenic; sometimes it is even protective. According to Denis and Obin [7] (paraphrasing Tolstoy), lean people seem to be mostly alike, whereas obese people are different, each in their own way. It seems that both obesity and cancer are heterogeneous, raising the question: what is “malignant” obesity?

Visceral adipose tissue (VAT), as indicated by central obesity, is thought to be pathogenic, whereas subcutaneous adipose tissue (SAT) is not [8]. In an interesting experiment, colonic adenoma-prone mice were divided into three groups—ad libitum fed, visceral fat removed with ad libitum feeding, and visceral fat removed with caloric restriction—and then compared. The adenoma rate was reduced by the removal of visceral fat but not by caloric restriction, indicating an independent effect of visceral fat on tumorigenesis [9].

Major studies, including the IARC report, used the body mass index (BMI) criteria for defining obesity. Although BMI is a good measure of total body fat mass, it is an inaccurate measure of clinically relevant obesity. VAT is better measured by other parameters, such as waist circumference (WC) and waist-to-hip ratio (WHR) or by computed tomography (CT) scanning [10]. An often quoted article explains how WC, not BMI, explains obesity-related health risk [11]. A recent meta-analysis suggested that central obesity measured by WC, not by WHR, is associated with modestly increased risk of both pre- and post-menopausal breast cancer, independent of general obesity [12]. More specifically, WHR adjusted for BMI appears to give a better measurement of clinically relevant obesity [13]. The pitfalls of using such measurements were discussed in a recent review [14]. A caveat is that our knowledge of clinically relevant obesity is from studies on metabolic syndrome (MetS) and insulin resistance; it is unclear whether ORCs have the same etiopathogenesis, and therefore it is unclear if these data are applicable. Researchers need to reach consensus on a more accurate definition and criteria for “malignant” obesity.

It is possible that excess adipose tissue is actually good for the body, indicative of adequate storage capacity for excess (and potentially lipotoxic) free fatty acid. The disease-protective effects of lower body fat have been summarized [15]. Surprisingly, the role of VAT as the pathogenic fat deposit has not yet been settled. Among the components of VAT, mesenteric fat may be more important than omental fat in metabolic dysregulation [16]. It is possible that the association between metabolic dysfunction and visceral obesity could be due to underlying processes that predispose to visceral obesity and metabolic dysfunction, rather than constituting a direct causal relationship [17].

An alternative hypothesis is that lack of adequate storage facility leads to ectopic deposition of fat in the liver and skeletal muscle, which causes insulin resistance and MetS [18]. Indeed, ectopic liver fat (ELF), which causes non-alcoholic fatty liver disease (NAFLD), predicts for and appears to be a precursor of MetS [19]. There are strong associations between obesity, MetS, and cancers [20]. Is it time to shift focus from VAT to ectopic fat as the risk factor for ORCs? (Or, in other words, does ELF cause ORC?) NAFLD is associated not only with liver cancers but also with extra-hepatic cancers [21]. Fatty liver releases “hepatokines” such as fetuin-A and FGF21 with significant roles in carcinogenesis; these roles are being investigated [22, 23].

It remains to be determined whether ectopic fat deposition, as measured by fat in the liver or by serum biomarkers such as alpha-ketoglutarate [24], will provide a more accurate measurement of “malignant” fat than VAT. Contradictory data exist. Conditions such as lipodystrophy, where there is absence of connective tissue fat with compensatory NAFLD and insulin resistance, do not appear to increase the risk for cancers (Dr. Abhimanyu Garg, personal communication). Additionally, African Americans are prone to obesity and ORCs but have a lower incidence of NAFLD, whereas the reverse is seen with Hispanics, who have a higher incidence of NAFLD but fewer cancers [25]. Central obesity is a stronger risk factor for cancer in Asians than in African Americans [26].

Therefore, factors that determine the distribution of fat in the deposits are relevant, and this appears to be coded in our genes. Evolutionarily speaking, in people from temperate climates, fat is distributed in the subcutaneous tissue where it acts as protection against cold (more SAT); if fat were distributed in the same way in people from tropical regions, it would cause overheating. In possibly the same way that the camel stores fat in its hump, people from tropical regions tend to store fat in the abdominal region (more VAT).

**Fig. 1:**
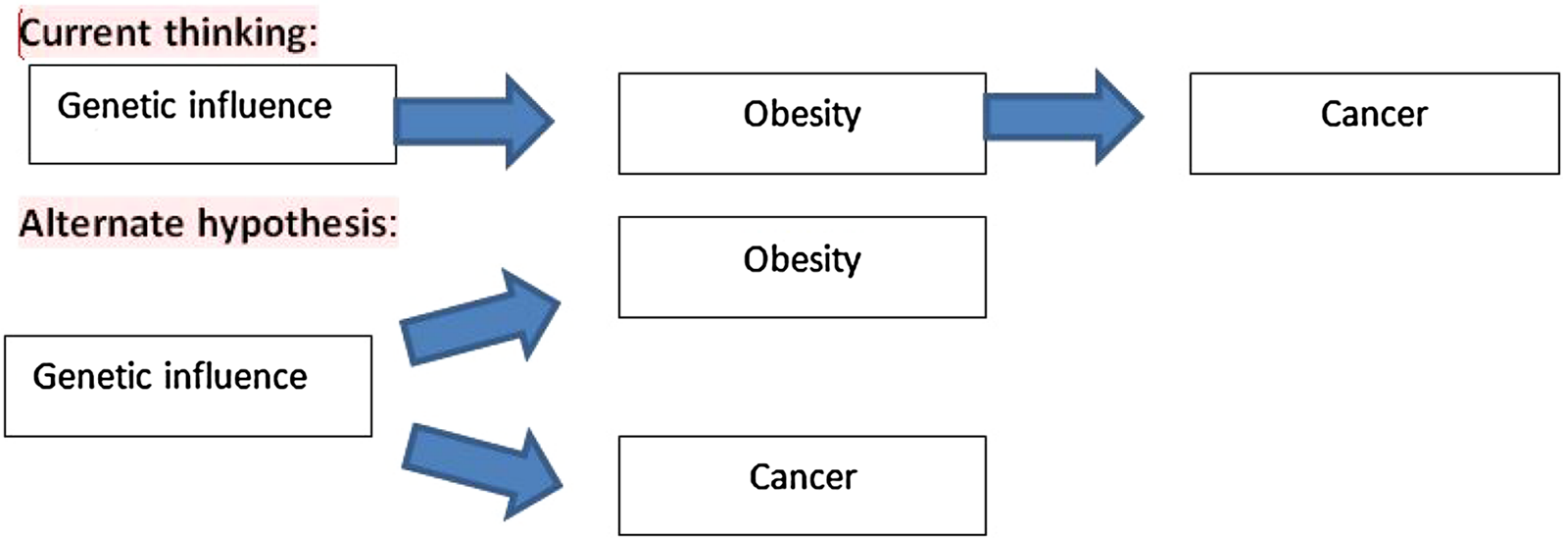
Relation between obesity and cancers: an alternate view

Clearly, the pattern of fat distribution is more important than the absolute amount of stored fat. Distribution of fat over the body is controlled by an array of “fat distribution genes.” An intriguing possibility is that people inherit a pattern of fat distribution genes that makes them susceptible, simultaneously, to central obesity and cancers; therefore, both fat and cancers are related but have independent outcomes. This is in line with current ideas on the causes of obesity [27] (Fig. 1). Transcriptional profiling of gluteal and abdominal fat has identified an extensive list of differentially expressed developmental genes, including members of the homeobox (HOX) family, HOX-domain-encoding genes (for example, *SHOX2* and *IRX2*), and T‑box genes (for example, *TBX15* and *TBX5*) [28]. These genes are known as transcriptional regulators and are involved in early embryonic development, body patterning, and cell specification. Interestingly, these genes are also implicated in the oncogenic process; that is, “fat distribution genes” are also oncogenic [29].

A genome-wide association study (GWAS) meta-analyses of traits related to waist and hip circumferences in 224,459 people identified a list of genes suspected to be involved in body fat distribution; these include familiar genes such as *SMAD6*, *BMP2*, *BCL2, PPARG*, and *VEGFA*, which are known to be involved in carcinogenesis [30]. Genes such as *HOXC8*, *HOXA5*, *TBX1*5, and *SFRP2* are expressed higher in VAT than SAT, and have also been implicated in carcinogenesis [31]. The exact relation needs to be further clarified; however, it does raise the possibility that both central obesity and ORCs are products of an early single event.

Approximately 10%–25% of obese people and a fraction of morbidly obese people do not have metabolic disturbances [32]. These “metabolically healthy, obese” (MHO) people are insulin sensitive and have normal blood pressure, a favorable lipid profile, a lower proportion of visceral fat, less liver fat, and a normal glucose metabolism, despite having an excessive amount of body fat. At the opposite end of the spectrum is a subset of normal weight people who suffer from metabolic disturbances that are characteristic of obesity (known as “metabolically obese but normal weight”). Analysis of the Framingham Heart Study of adults who were obese and glucose tolerant revealed that potentially MHO people have a much lower risk of obesity-associated cancer than those who are obese, glucose-intolerant, and potentially “metabolically unhealthy, obese” [33]. The implications are that obesity is not a default pro-carcinogenic state and that cancers can be uncoupled from obesity.

The brd2-deficient mouse (in which the level of *brd2* gene expression is decreased; total knockout is lethal) appears to be a good model to study this possibility [34]. This mouse is obese with hyperinsulinemia but is metabolically healthy, appears to be tumor-free, and has a long life (up to 20 months). Therapeutic blocking of this gene or its pathway could be an option to be obese but healthy.

To add to the complexity, adverse sequelae of obesity can be modified by other risk factors, such as ethnicity, sex, age at which the person became obese, and especially height [35]. Compared with Caucasians at the same BMI, South Asians have approximately three times the risk and Chinese have approximately twice the risk of developing type 2 diabetes [36]. The Framingham Heart Study indicated that being overweight during adolescence may be a more significant predictor of colorectal cancer risk than being overweight during adulthood [36]. Moreover, the association of height with cancer is particularly strong [35] and is one more reason why BMI is such an unreliable measurement of obesity.

**Implications:** There is an urgent need to better define oncogenic fat (SAT vs. VAT vs. ectopic fat) and to measure it (BMI vs. WC; CT scan vs. biopsy vs. circulating markers of NAFLD) to identify a homogenous at-risk population that can be targeted for interventions. However, such criteria should take into account ethnic differences [36]. Data from animal studies (brd2-deficient mice) and human studies (MHO people) suggest that cancer is not an invariable result of obesity and that future interventions could uncouple them.

The IARC identified several cancers that are associated with obesity [5], but it did not specify which subtype. There are many studies on the morphologic subtypes associated with obesity [37–41] (representative sample in Table 1).

**Table 1:** Morphological subtypes of cancers related to obesity

No.	Site	Morphologic subtype
1.	Endometrium	Type I-endometroid
2.	Colon	Left sided
3.	Breast	Post-menopausal, luminal type, triple negative
4.	Kidney	Clear cell renal cell cancer
5.	Ovary	Low grade serous

Summarized from references [37–41]

However, the current emphasis is on molecular classification of cancers. Which molecular subtype(s) are linked to obesity? The new field of molecular pathological epidemiology (MPE) may provide some answers [42].

Studies linking obesity to cancer have been performed at the genetic and epigenetic levels. At the genetic level, alterations in single gene expression and gene pathways have been observed, and some molecular signatures have been generated. Alterations restricted to single genes are rarely reported. Low levels of *FASN* (the gene for fatty acid synthase) are seen in obesity-related colon [43] and renal cancers [44]; additionally, a relation has been observed in post-menopausal breast cancer with higher BMI [45]. FASN is associated with poor prognosis, and low levels in ORCs could explain the “obesity paradox” [39].

Although tempting, it is unlikely that single gene alterations will explain the etiology or identify environmentally derived cancers; differences are more likely to lie at least at the pathway level [46]. The data generated by various studies linking obesity to molecular subtypes are contradictory, possibly related to differing criteria of obesity. For example, many studies of colorectal cancer did not show consistency on which pathway [the CpG island methylator phenotype (CIMP), the chromosomal instability (CIN), or the microsomal instability (MSI) pathways] is dysregulated. Another study showed that obese patients were more likely to be microsatellite stable (MSS) with a deficient mismatch repair (dMMR) rate of 10.3%, which was significantly lower than the dMMR rates of 17.1%, 17.4%, and 21.8% in overweight, normal weight, and underweight BMI categories, respectively [47]. Overall, it appears that, at the molecular level, obesity-related colorectal cancer is CIMP-low [48] and non-MSI, with altered beta-catenin (CTNNB1) and p53. However, findings could vary depending on sex and definition of obesity (BMI vs. WHR or WC), as shown by the Malmö Diet and Cancer Study [49].

The link between obesity and prostate cancer is an example of contradictions that occur when all molecular subtypes are grouped together. A meta-analysis showed that the link between obesity and incidence of prostate cancer is weak [50]. Recent evidence suggested that obesity is specifically associated with a reduced risk of developing androgen-responsive T2E (TMPRSS2:ERG) fusion-positive tumors [51]; this fusion gene is found in 50% of prostate cancers, and this could be related to lower levels of androgen in obese men. Therefore, linkage studies should be specific not only about the type of obesity but also about the molecular subtype of cancer.

One study showed that obesity and physical inactivity were associated with a higher risk of CTNNB1-negative colorectal cancer [52]. This is consistent with findings in studies of the classical endometrioid endometrial cancer in obese women, which are usually negative [53]. A recent study analyzing The Cancer Genome Atlas (TCGA) data concluded that gene expression in endometrial cancer is related to BMI [54].

Current research using genomic profiles by techniques such as microarrays have generated molecular subtypes for most cancers (notably by TCGA). These molecular subtypes have been associated with response to treatment and survival (outcomes which are variable and strongly influenced by treatment). It is curious that less effort has gone into defining subtypes based on etiology (which is fixed). Next-generation sequencing technology enables us to observe DNA-sequence-level effects of well-known mutagens, such as ultraviolet radiation and tobacco smoke, as well as endogenous mutagenic processes, such as those involving activated DNA cytidine deaminases (APOBECs), and generate specific mutation signatures. However, so far, no obesity-associated signature has been demonstrated. GWASs conducted by multiple consortiums have generated lists of single nucleotide polymorphisms (SNPs) associated with obesity. These SNPs were used to produce genetic risk scores as a surrogate of “true” (genetic) obesity and then used to link obesity with cancers. The results of these Mendelian randomization studies have been usually consistent with what we know from epidemiologic studies [38, 55, 56].

Obesity is more likely to alter the epigenome than the genome [57]. Diseases including cancers can arise out of alterations in the epigenome [58, 59]. Campión et al. [60] has identified epigenetically altered genes implicated both in obesity and in cancer. Many studies have focused on the altered epigenome of obese people in blood cells and adipose tissue [61], normal breast tissue [62], breast cancer [63–65], and endometrial precursor lesions and cancer [66]. There should be more such epigenome-wide association studies (EWASs) to generate molecular signatures across other ORCs and to see if commonality can be identified across these signatures, irrespective of tissue of origin.

Multiple genomic and epigenomic studies have been done with smoking, which is the other major risk factor for cancer. GWASs have shown gene expression signatures in normal tissue that consistently differentiated never-smokers from smokers [67]. It is thought that tobacco smoke (like other environmental carcinogens) is more likely to alter the epigenome, and EWASs have identified molecular signatures specific to tobacco smoke [68–71]. Some changes are apparently precursor lesions, appearing before the onset of lung cancer [72]. It is not unreasonable to expect similar signatures for ORCs.

It would be interesting to know why some organs remain free of ORCs. For example, the brain is apparently resistant (related to the blood–brain barrier?), and finding the underlying protective mechanism could be useful. Other “protected” organs include the rectum, small intestine, urinary bladder, testis, and connective tissue. Lung cancer is another, but this could be due to the competing stronger risk factor of smoking.

Smoking is known to be associated with cancers developing at multiple sites, such as the head and neck, lung, and bladder [73, 74], but similar data are lacking in obese cancer patients. An early study based on Surveillance, Epidemiology, and End Results data suggested that multiple primary associations were compatible with the existence of common etiologic dietary elements, but this study did not specifically look at obesity [75].

**Implications:** Generating genome/epigenome expression profiles based on etiology rather than natural history will provide a homogenous group of ORCs, which can be further studied to clarify the mechanisms of carcinogenesis and targets for interventions.

### Question 2

Through which factors/genes/signaling pathways does adipose tissue influence tumorigenesis?

How does (a specific type of) obesity cause (a specific type of) cancer? [76].

Some explanations for “adiponcosis” are site-specific—for example, esophageal adenocarcinomas due to reflux and Barret’s esophagus; breast cancer due to raised estrogens generated from fatty tissue via aromatase enzyme; liver cancer due to NAFLD; and gallbladder cancer due to higher incidence of gallstones. A recent study suggested that an excess of calories suppress *GUCY2C* signaling (a tumor suppressor gene), resulting in intestinal tumorigenesis in obesity [77, 78]. However, no single unifying explanation has been found to be satisfactory.

Dietary components (such as aflatoxin B) can be carcinogenic, but such cancers occur irrespective of a person’s nutritional state. Unsaturated fatty acids may directly alter oncogenic pathways [79]. Direct modification of oncogenic pathways by microRNA ingested from milk is a postulated mechanism, but whether adequate amounts are absorbed is a matter of debate [80].

The two postulated mechanisms by which VAT can cause cancer are metabolic dysregulation and chronic inflammation [81]. VAT is associated with higher levels of insulin and insulin-like growth factor 1 (IGF-1) [82] and releases factors involved in metabolic dysregulation, such as adipokines (leptin and adiponectin). Major components of the inflammation pathway are cytokines such as tumor necrosis factor (TNF)-alpha, interleukin (IL)-1-beta, IL-6, IL-1 receptor antagonist, soluble TNF-alpha receptor, and C-reactive protein (the “secretome”) [83]. It is also unclear whether the “secretome” has a paracrine effect or endocrine. For example, does the fat around the breast (SAT) induce breast cancers, or is it an endocrine effect of cytokines from VAT? Evidence exists for both mechanisms [84–86].

The mitogenic and potentially pro-carcinogenic effects of insulin [87] have been hypothesized but unproven for decades [88]. The brd2-deficient mouse referred to earlier is obese and hyperinsulinemic but lives tumor-free [34]. In contrast, studies in “fatless mice” (A-Zif/F1) suggest that adipokines may not be as important as high levels of insulin, IGF-1, and inflammation, and activation of the phosphoinositide 3-kinase (PI3 K)/Akt pathway is central to increased cancer risk [89].

The relation between cancer and inflammation has been well summarized by Colotta et al. [90]. Up to 40% of VAT is composed of infiltrating macrophages that are thought to cause a low-grade “chronic inflammatory state,” which is pro-carcinogenic. Reactive oxygen and nitrogen intermediates are obvious inflammation-generated candidates causing DNA damage [91]. While there is evidence linking inflammation and cancer [92], as seen in organ-specific inflammatory disorders such as ulcerative colitis, the evidence is weaker for systemic inflammatory disorders such as autoimmune diseases. For instance, in a large cohort of systemic lupus erythematosus patients, the overall risk of cancer was only marginally increased to 14%, and the spectrum was different (significantly only for cancers of the vagina and liver and less so for cancers of the lung, kidney, and thyroid; cohort members had actually lower risks of cancers related to obesity, such as breast, uterus, cervix, and prostate cancers) [93]. Indeed, chronic syphilis (another inflammatory state) is traditionally thought to be protective against cancer. Colotto et al. [90] argues that it remains uncertain whether chronic inflammation per se´ is sufficient for carcinogenesis. A potential candidate is galectin-3, which is secreted by macrophages infiltrating adipose tissue. Galectin-3 is an important regulator of diverse functions that are critical in cancer biology, including apoptosis, metastasis, immune surveillance, molecular trafficking, mRNA splicing, gene expression, and inflammation, and it has been recently identified as a critical factor in insulin resistance [94].

Whatever the active components of the malignant “secretome” (metabolic vs. inflammatory), it will have to act through oncogenic signaling pathways. Those implicated include the phosphatidylinositol 3-kinase/protein kinase-B/mammalian target of rapamycin (PI3 K/Akt/mTOR), the mitogen-activated protein kinase (MAPK), the signal transducer and activator of transcription 3 (STAT3), and the NK-κB pathways. The strongest evidence exists for the PI3K/AKT/mTOR pathway, which is the proliferative pathway implicated in many cancers. For instance, many of the factors up-regulated in the obesity signal via the PI3K pathway. These include leptin, IL-6, insulin, TNF, and IGF-1 [95]. PI3K activity is increased in diet-induced obesity in mice [96], and tumor growth can be abrogated by metformin, which reduced AKT levels [97]. Tumors that are resistant to dietary restriction have constitutive activation of the PI3K pathway [98]. The evidence for involvement of PI3K pathway in obesity, inflammation and metabolic dysregulation has been summarized by Beretta et al. [99].

A couple of issues are worth speculating on. Recent studies have shown that obesity can be inherited through epigenetic changes that affected the parents (transgenerational inheritance of obesity) [100]; this raises the question of inheritance of ORCs by similar mechanisms [101]. Many cancers have familial clustering but do not have a clearly identifiable gene candidate; these are usually attributed to low-penetrance genes or SNPs. But such familial clustering could possibly be due to similar epigenetic mechanisms of transgenerational inheritance.

The second issue is, does energy imbalance act early or late in carcinogenesis? Carcinogenesis, especially when caused by environmental influences, is a multistep process, and it would be interesting to know whether obesity is the initiator or promoter of cancers.

**Implications:** It is essential to know which are the pathogenic elements of the secretome and through which signaling pathway(s) they act. Such information is vital from a preventive and therapeutic perspective. The possibility of transgenerational inheritance of obesity raises the possibility that a person could be susceptible to cancer just because their parents were obese; more epidemiologic studies need to be oriented towards this link.

### Question 3

Are there common (overlapping) genetic loci that make individuals susceptible to obesity, metabolic syndrome, and cancers?

In other words, is there a genetic susceptibility to obesity, and are these people prone to cancers? Pigeyre et al. [102] summarized the current evidence regarding the genetic basis of obesity. Overgrowth syndromes are known to have neoplastic susceptibility [103]. Examples include WAGR (Wilms tumor), Beckwith-Weidemann syndrome (embryonal tumors), and PI3K-related syndromes (multiple tumors). This is consistent with the possibility mentioned above that a common genetic factor could predispose a person to both obesity and cancer.

However, people with polymorphisms of genes such as *FTO* or *MC4R*, which are associated with risk of obesity, have not been found to have a higher risk of colorectal [104] or endometrial cancer [105] compared with those without these polymorphisms, although a higher risk of breast cancer has been noted [106].

Epigenetic factors strongly influence susceptibility to obesity. Both intrauterine growth restriction with low birth weight, and maternal high-fat diet with high birth weight (and, to add to the complexity, low birth weight with accelerated catchup growth due to postnatal nutrition) are associated with adult obesity, insulin resistance, metabolic syndrome, NAFLD, and diabetes [107]. In sharp contrast to the large amount of data regarding metabolic perturbations, scant data exist on whether these types of obesity (assuming that different epigenetic mechanisms are at work) are associated with increased risk of cancer. Mice models suggest that the offspring of rat dams that receive a protein-restricted (low protein) diet throughout pregnancy and lactation develop mammary tumors more quickly; added nutrition simulating accelerated catchup growth increased this risk [108]. Consistent with these experimental findings, prenatal exposure to starvation increased the risk of breast cancer in women of the Dutch Famine cohort [109]; they also had higher cancer-related mortality [110]. Data also shows that the *offspring* of mice on a high-fat diet have a higher risk of mammary tumors. This is a cause of concern as the ill-effects of the ongoing obesity pandemic [111] could be amplified in future generations.

Not all obese people develop cancers, and not all tissues in obese people are susceptible to ORCs. Cancer susceptibility in obese people appears to be strongly related to fat distribution (whatever the mechanism), but tissue susceptibility to ORCs is a matter of speculation. In line with the recent speculation of “bad luck” cancers, ORCs have been attributed to increased stem cell proliferation [112, 113] and to differential expression of insulin and IGF receptors on tissues [114].

**Implications:** Identification of genetically and epigenetically susceptible “at-risk” individuals is a priority for targeted early intervention. It is unclear at this point whether these tests will be in the form of genome assays or specific serum biomarkers; the ethical implications of these tests are unclear as well. The type of intervention is also not clear but encouraging data are available on drugs such as metformin [115] and on more aggressive approaches, such as bariatric surgery, in reducing the risk of cancers [116].

### Question 4

Are there precursor lesions of ORCs, and will their identification help alter the natural history of such cancers?

The decline in cardiovascular mortality is due to identification of early events on the road to potentially fatal events, such as myocardial infarction and stroke (such as diabetes, hypertension, and hyperlipidemia), and aggressive treatment of the same. Unfortunately, precursor lesions (such as ductal carcinoma in situ in breast cancer, cervical intraepithelial neoplasia in cervical cancer, and polyps in colon cancer) detected during screening procedures need aggressive treatment, which is sometimes as morbid as the treatment of established cancer.

Can we detect precursor lesions for ORCs? There are tantalizing glimpses of future possibilities. Altered pattern of epigenetic changes occur even before the appearance of cancer. Biopsy of colonic mucosa of healthy women showed that methylation in distal mucosa increases with higher BMI [117]. Similarly, FASN has been shown to be overexpressed in the rectal mucosa of obese people, especially men, and has been proposed as a marker for colonic neoplasia that is present elsewhere [118]. Both “normal” endometrial glands and endometrial complex atypical hyperplasia in obese women showed low levels of p27 [119]. Other reported changes in precursor lesions include low STMN1 and high KRAS expression; KRAS mRNA expression was highly associated with high BMI [66].

However, the current data have two major limitations. One, the sensitivity, specificity, and reproducibility of these lesions have yet to be proved relevant for clinical use. Two, as shown, a universal precursor lesion that is applicable across all tissue types is yet to be demonstrated.

It is not possible to biopsy multiple organs-at-risk in obese people in the hope of finding precursor lesions. It is possible that the developing technology of “liquid biopsies” (detection of circulating tumor cells, cell free DNA, or exosomes) will prove useful in the future to screen for ORCs [120]. However, if precursor lesions are to be detected in people at high risk, we need to know the specific gene signature identifying precursor lesions of ORCs, preferably a universal signature of an ORC-related epigenome in circulating healthy or dysplastic epithelial cells. Positive liquid biopsies in such individuals would be an early warning and set the stage for interventions. Altered methylation pattern has been detected in the lymphocytes of obese people [121], but more studies are needed to identify precursor circulating epithelial cells. Several companies, such as Grail (by Illumina), are working to identify circulating tumor DNA by liquid biopsy as a method of screening for early cancer.

**Implications:** The ideal precursor lesion would (1) be detectable in circulating healthy or dysplastic cells on “liquid biopsies;” (2) be a molecular signature involving multiple genes (a single mutation is unlikely to explain the link, considering the heterogeneity of issues involved); (3) be universal (cutting across tissue types); (4) have a possible signature that involves alterations to the epigenome; (5) be indicative of pathways involved in metabolism and cell proliferation, possibly immunity and inflammation; and (6) contain markers for tissue of origin to localize the organ-at-risk (to avoid biopsying multiple at-risk organs).

As of now, no such biomarker exists; and from a study of the literature, there appears to be no effort to find one.

## Conclusions

Obesity is believed to be a major cause of cancers. However, heterogeneity of types of obesity and types of cancers, with competing causes, has resulted in a minefield of conflicting data. The cause of obesity itself is being debated [27]. Accumulating evidence shows that the paradigm OBESITY = CANCER is inaccurate.

The obesity pandemic is projected to progress, and because of this, ORCs are expected to become more common [122, 123]. ORCs are a research priority. It is estimated that overweight and obesity accounts for 14% of all cancer deaths in men and 20% of those in women [124]. Teasing out a homogenous group of cancers that are etiologically related at the molecular level (irrespective of primary site) is vital; this can be achieved by stricter definition of obesity in molecular level. We need to improve our understanding of how epigenetic events affect a person’s predisposition to obesity and how obesity affects the epigenome [125]. In response to the summoning of *Chinese Journal of Cancer* for collecting key questions in cancer research and clinical oncology [126] 4 questions are presented here. Future studies are expected to explore the following directions:

- A clearer definition of pathological obesity that can be measured easily.
- Molecular profile of ORCs based on such criteria.
- Expanded research into the causative mechanism(s).
- Research into uncoupling obesity from cancer and MetS by targeting the responsible genes.
- Repurposing of drugs currently used in MetS to test their efficacy in the prevention and treatment of ORCs.
